# A leakage-controlled multi-compartment benchmark of fluid biomarkers for predicting mild cognitive impairment to Alzheimer’s disease conversion: plasma, CSF, and high-plex omics in a single cohort

**DOI:** 10.21203/rs.3.rs-10580655/v1

**Published:** 2026-08-05

**Authors:** Ivan Javier Cangas Caiza

**Affiliations:** Yachay Tech University, Ecuador

**Keywords:** Alzheimer’s disease, mild cognitive impairment, biomarkers, plasma, CSF, proteomics, conversion prediction, cross-validation, data leakage

## Abstract

Approved disease-modifying therapies have made it a practical priority to predict, early and at low cost, who among people with mild cognitive impairment (MCI) will progress to Alzheimer’s disease (AD) dementia. The candidate biomarkers sit across biological compartments that differ sharply in invasiveness and cost: blood plasma, cerebrospinal fluid (CSF, via lumbar puncture), and high-plex “omics” assays. Once data leakage in feature selection is controlled, how much each compartment truly contributes to predicting MCI-to-AD conversion is an open question, and answering it is the point of this benchmark.

Using the Alzheimer’s Disease Neuroimaging Initiative (ADNI), we benchmarked prediction of MCI to AD conversion across four clinical compartments (demographics, classic plasma, plasma mass-spectrometry, classic CSF) and four omics compartments (plasma metabolomics, plasma lipidomics, CSF metabolomics, CSF SOMAscan proteomics). Validation used 5-fold cross-validation with bootstrap 95% confidence intervals and nested, in-fold feature selection to keep leakage out.

The best single compartment was classic plasma (p-tau217/NfL/GFAP), which reached an AUC of 0.785 (0.71–0.85); CSF proteomics (SOMAscan) came close behind at 0.758 (0.71–0.80), and classic CSF followed at 0.747 (0.71–0.79), whereas plasma metabolomics and lipidomics sat near chance at 0.51–0.60. When the sample is narrowed to the 159 subjects who had complete data on all three, the comparison gets sharper still. Plasma, in other words, is where most of the usable information sits: put it on top of demographics and the AUC climbs from 0.578 to 0.769, an NRI of +0.79. CSF, on top of all that, is worth only another +0.03 of AUC, a gain too small to lean on. APOE4 keeps predicting conversion on its own, at an HR of 1.83 per allele (p=0.008). So, to say it plainly: a blood-based panel already carries most of what is needed to predict MCI to AD conversion, and CSF adds only a little on top of it. Read for practice, the results support a staged, blood-first screening strategy, and they put a concrete number on what each additional compartment contributes once overoptimism is accounted for.

## 1 Introduction

The approval of disease-modifying therapies for early Alzheimer’s disease (AD) has turned early, non-invasive identification of at-risk people into a practical priority. Concretely, the aim is to spot the individuals with mild cognitive impairment (MCI) who are most likely to progress to AD dementia, and to spot them while the window for intervention is still open. Blood and cerebrospinal fluid (CSF) biomarkers have moved to the centre of this effort, and that is where they belong. They sit alongside, and sometimes instead of, amyloid and tau positron emission tomography and the invasive lumbar puncture that used to be the only route we had.

The trouble is that the biomarkers live in different biological compartments, and those compartments are far from equal in invasiveness and cost, which is the whole problem in a nutshell. Plasma costs little and is easy to draw, which is exactly why it appeals; the flip side is that its analytes arrive diluted, partly cleared by the periphery on the way. CSF sits nearer to the brain tissue, but reaching it calls for a lumbar puncture, so it is not something you do lightly. The high-plex “omics” assays, SOMAscan proteomics, metabolomics, lipidomics, measure thousands of analytes in one go, and they pay for that reach in money and technical effort. A sound clinical pathway really comes down to a single practical question: how much predictive value does each compartment add, for its inconvenience? That is the trade-off, and most of the literature has simply not laid it out in comparable terms.

Most prior reports concentrate on a single biomarker or a single winning compartment. Recent large-scale CSF proteomics studies have tied synaptic protein signatures to cognitive decline [2, 3], but a systematic, leakage-controlled comparison of the marginal value of each compartment for the specific task of MCI to AD conversion, weighing performance against accessibility, remains largely missing. The reason is partly methodological, in the form of data leakage in feature selection. When predictive features are chosen using the full dataset and cross-validation runs afterwards, out-of-sample performance is overestimated, and our own earlier proteomics results were inflated by this mechanism, returning to a credible range only after nested, in-fold feature selection.

A separate, well-established result concerns diagnosis: plasma p-tau217 performs equivalently to CSF p-tau217 for biomarker-defined AD [1], and later reports have reproduced it [5, 6, 7]. The parallel with diagnosis, though, has to be drawn carefully, because the two tasks are not really the same. The equivalence concerns diagnostic classification, in other words amyloid status and biological AD under the AT(N) framework [9]; it generally pits plasma against CSF and nothing else, and most studies settle it in separate datasets or separate cohorts. Conversion is not the same task, and it is the harder one. In its multi-compartment form, our question is how much predictive value each biological compartment contributes to MCI to AD conversion, as opposed to diagnosis, and we ask it within a single cohort, under one leakage-controlled methodology, scored with clinical-decision metrics such as NRI and decision-curve analysis. Multi-omics blood studies have put forward candidate biomarkers [8], but none, to our knowledge, brings CSF, proteomics, metabolomics, and lipidomics into the same head-to-head benchmark for conversion.

Here we present such a benchmark, built on ADNI, for MCI to AD conversion under strict leakage control. We report, first, the AUC of each compartment on its own, and second, the marginal gain of adding compartments in a common-subject, head-to-head analysis quantified with NRI and decision-curve analysis. Our plasma-versus-CSF result is offered as a replication of the published equivalence, carried here into the longitudinal conversion setting and into a context that holds multiple omics compartments side by side.

## 2 Materials and Methods

### 2.1 Data and cohort

All data come from the Alzheimer’s Disease Neuroimaging Initiative (ADNI; adni.loni.usc.edu) [10]. Every participant entered the analytical cohort with a baseline diagnosis of MCI, and for each one the outcome was known: progression to AD dementia during follow-up, or censoring. We built one row per subject at baseline. Cohort sizes for each analysis are reported in the Results; further detail on data harmonization lives in the project repository.

### 2.2 Compartments

We defined eight compartments as follows:

- **Demographics:** Age, sex, education
- **Classic plasma:** plasma p-tau217, neurofilament light (NfL), glial fibrillary acidic protein (GFAP) [4].
- **Plasma mass-spectrometry A***β*: plasma A*β*42/A*β*40 ratio by mass spectrometry.
- **CSF Classic:** CSF A*β*42, total tau (t-tau), phosphorylated tau (p-tau181).
- Plasma metabolomics (Nightingale NMR).
- Plasma lipidomics (NDuke Q500 FIA).
- CSF metabolomics (Cruchaga lab).
- CSF proteomics (SOMAscan 7k)..

### 2.3 Model and validation (leakage control)

Logistic regression was the workhorse model throughout, run under 5-fold stratified cross-validation; within each training fold, features were standardized with robust scaling, and nothing from outside that fold was allowed to leak in. We did try the non-linear routes first, but on these same cohorts random forest and XGBoost simply failed to outdo logistic regression, so the interpretable linear model carried the day, which is what we wanted anyway. For the high-dimensional omics compartments (C5–C8), feature selection itself had to be nested within each fold to stay honest: analytes are ranked on the training fold, and only the top-k are what feed that fold’s test model. Do it the other way, selecting on the full dataset up front, and the out-of-sample numbers get a free boost, which is exactly the kind of leakage that took an earlier SOMAscan estimate from a true 0.737 to 0.773 before it was caught and corrected.

AUC confidence intervals rest on 1,000 bootstrap resamples, and sensitivity and specificity come back at the Youden-optimal threshold.

### 2.4 Marginal-gain (head-to-head) analysis

To compare compartments fairly on the same subjects, we restricted the analysis to the subjects with complete data for demographics, plasma, and CSF (n = 159; 53 conversion events). We fitted nested models: - M1 demographics; - M2 M1 + plasma classic; - M3 M2 + CSF classic.

We report the AUC and its 95% CI for each model, the change in AUC (AUC) between adjacent models, the continuous net reclassification improvement (NRI), and decision-curve analysis (net benefit) across clinically relevant risk thresholds (0.20–0.50).

### 2.5 APOE4 analysis

APOE4 status (number of *ϵ*4 alleles) was available in a subset of MCI participants (n = 295). We fitted a Cox proportional-hazards model of conversion on APOE4 dosage, adjusted for age and sex, with time to event defined as time to conversion (events) or time to censoring (non-events).

## 3 Results

Continuous variables are mean ± SD (n with non-missing value). Categorical variables are n (%). Conversion status available for n=1543 MCI participants (416 converters, 1127 non-converters); individual biomarkers have smaller n because not all participants were assayed for every marker.

APOE4 dosage is the count of *ϵ*4 alleles (available in a subset); percentages are within each conversion group. Formal APOE4 inference is provided by the time-to-event Cox model in Results (HR 1.83 per *ϵ*4 allele, p=0.008), which incorporates censoring and follow-up time. p-values: Welch’s t-test (continuous), chi-square (categorical).

### 3.1 Compartment benchmark

Baseline characteristics of the analytic MCI cohort, stratified by conversion status, are shown in Table 1 (416 converters vs 1,127 non-converters). Converters were older and carried significantly more AD pathology at baseline (higher plasma p-tau217, lower A*β*42/40, lower CSF A*β*42, higher CSF t-tau and p-tau181), consistent with an enriched preclinical-to-prodromal profile.

**Figure 1:**
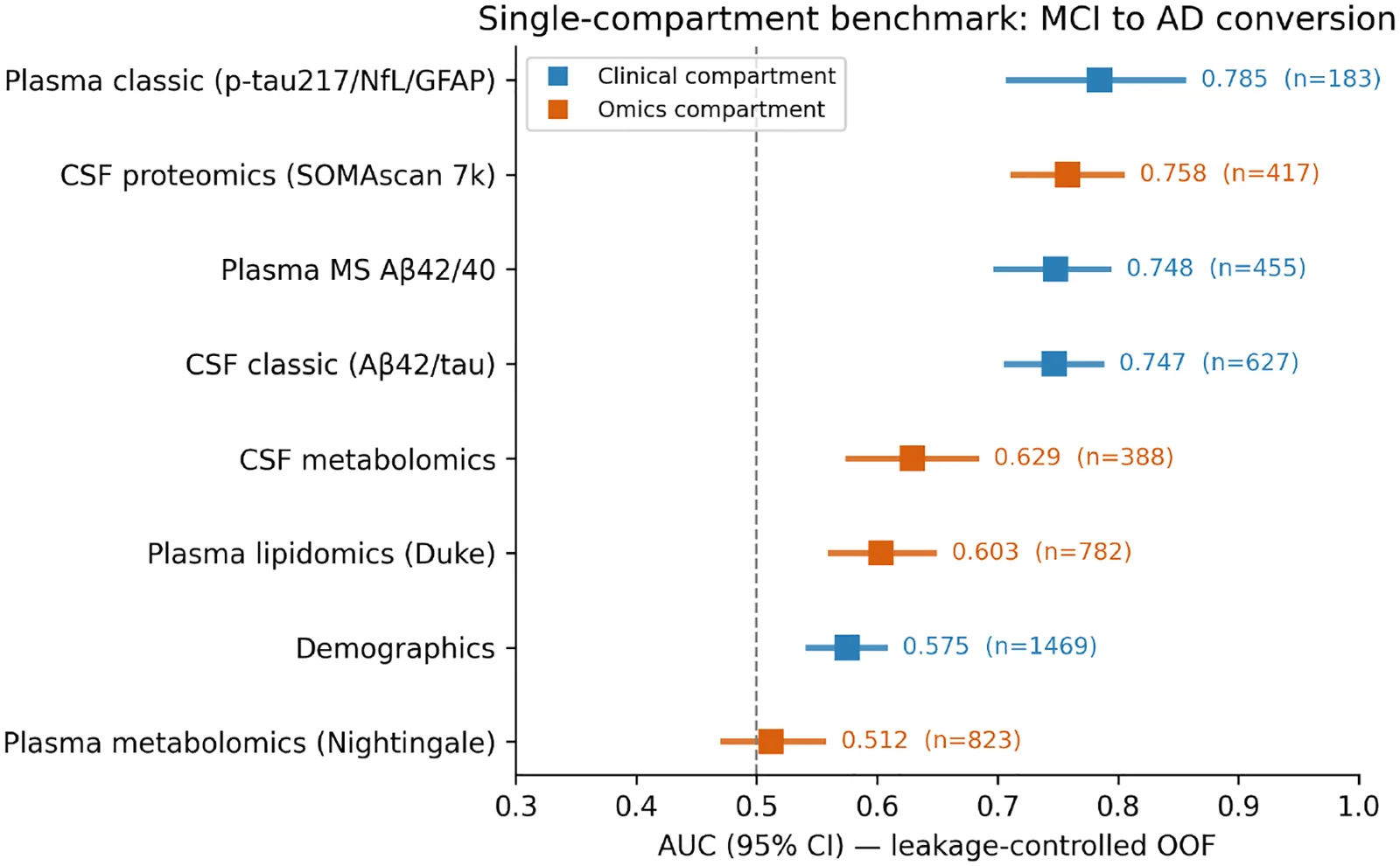
Single compartment benchmark: MCI to AD conversion. Leakage-controlled out-of-fold AUC with bootstrap 95% CI

**Figure 2:**
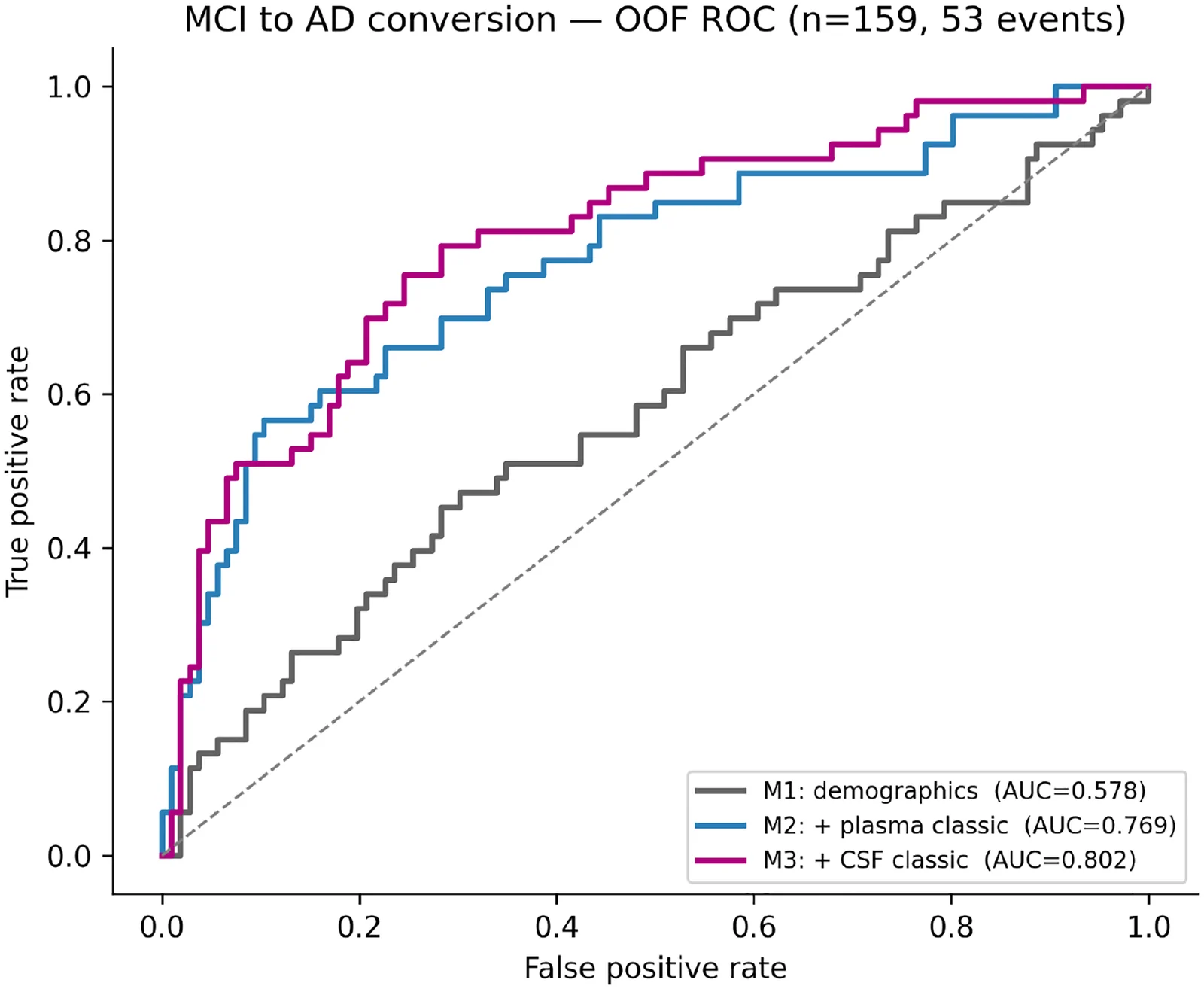
Out-of-fold ROC curves for nested models in the common-subject cohort (n=159, 53 conversion events).

Each compartment was evaluated in its own available subcohort (sample size differs across compartments; the head-to-head comparison is in Section 3.2, Figure 2). Figure 1 shows the single-compartment AUCs as a forest plot.

The classic plasma panel was the best single compartment. CSF proteomics did not outperform classic CSF, and both plasma omics compartments performed near chance.

### 3.2 Marginal gain in a common cohort

In n = 159 subjects with complete demographics, plasma, and CSF, the nested models yielded:

Adding the plasma panel to demographics produced the large gain (+0.19 AUC, NRI +0.79). Adding CSF on top of plasma produced only a marginal gain (+0.03 AUC, NRI +0.42), within the overlap of the confidence intervals. Figure 2 shows the corresponding out-of-fold ROC curves for the three nested models.

**Figure 3:**
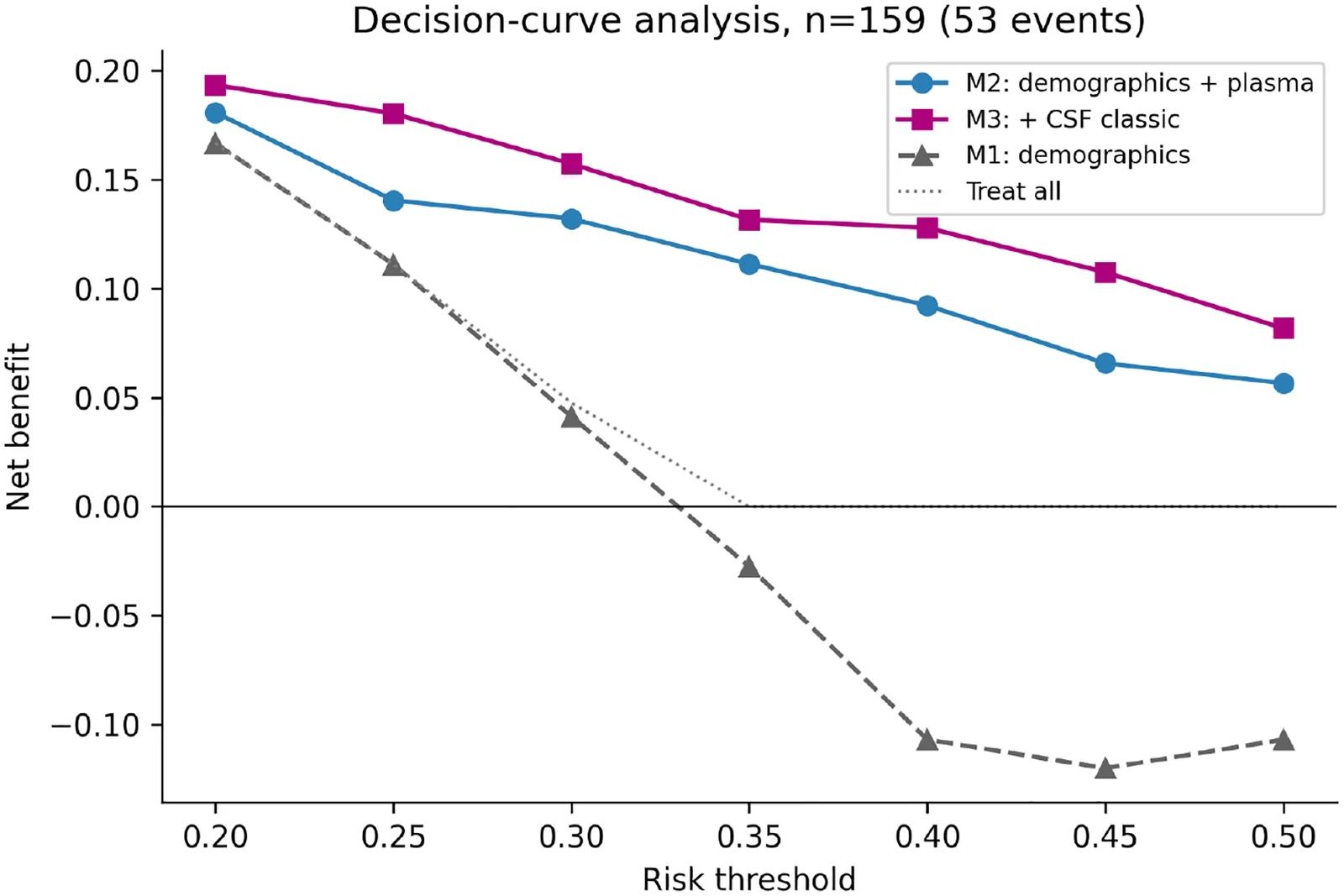
Decision-curve analysis for nested models; net benefit across clinically relevant risk thresholds

Decision-curve analysis showed monotonically increasing net benefit across models (M1 ¡ M2 ¡ M3) at all evaluated thresholds (0.20–0.50); the largest improvement occurred when adding plasma (Figure 3).

### 3.3 APOE4

APOE4 dosage was associated with conversion in a Cox model adjusted for age and sex (HR = 1.83 per *ϵ*4 allele, 95% CI 1.17–2.85, p = 0.008). Conversion rates rose with dosage: 12.3% (0 alleles), 18.1% (1), 18.5% (2).

## 4 Discussion

### 4.1 Principal findings

A single plasma panel measured at baseline already captured most of the predictive information we could extract for MCI to AD conversion. The strict head-to-head analysis makes the point cleanly: plasma accounted for nearly all of the improvement over demographics, while CSF, the more invasive and costlier compartment, shifted the numbers only marginally. The same panel also matched, and in places beat, a 7,000-analyte CSF proteomics assay at a fraction of the price; plasma metabolomics and lipidomics, for their part, stayed near chance. The accessible measurement held its own against the invasive one, which is exactly the finding that matters in practice.

### 4.2 Interpretation and clinical relevance

A blood-first, staged screening strategy for MCI to AD conversion risk follows from the numbers: start with the cheap plasma panel and keep CSF sampling in reserve, for the cases that actually need the extra discrimination. The decision-curve analysis supports that reading. Across the relevant thresholds the net clinical benefit of the plasma model holds up, and the gain from adding CSF stays modest. The field tends to ask which biomarker is best. The practical question raised here is instead how much predictive value each accessible compartment adds for its own cost and invasiveness, and that is the question health systems planning scalable AD screening most need answered.

### 4.3 Comparison with prior work

Large-scale CSF proteomics studies have reported synaptic-protein signatures tightly bound to cognitive outcomes; the CSF YWHAG:NPTX2 ratio, for instance, predicts conversion to dementia with hazard ratios around 2–3 [2, 3]. Our results agree with that line of work to the extent that CSF carries genuine signal. Where we differ is in the comparison that matters clinically: plasma versus CSF on the same subjects.

Our finding that a classic plasma panel performs comparably to, and adds little beyond, CSF replicates the established plasma p-tau217 = CSF equivalence reported for diagnosis [1]. The extension here is twofold: we bring that equivalence into the longitudinal conversion setting, and we hold it inside a head-to-head context that simultaneously includes high-plex omics compartments, namely CSF proteomics, plasma and CSF metabolomics, and plasma lipidomics. In this light the original contribution is not that plasma equals CSF. It is the multi-compartment, leakage-controlled benchmark itself, the systematic accounting of each compartment’s marginal value for conversion in a single cohort, which to our knowledge has not been carried out together for plasma, CSF, and six-plus omics profiles.

Not all blood multi-omics work lands where ours did, and it is worth saying why. Some studies report high diagnostic accuracy [8], yet our plasma metabolomics and lipidomics compartments came out near chance for conversion, at an AUC of 0.51–0.60, which is basically a coin flip. Take Gómez-Pascual et al. as an example: they brought plasma lipidomics together with proteomics in a machine-learning model of MCI-to-AD progression and turned up key metabolites and proteins, so they clearly had signal. The discrepancy with our near-chance lipidomics is probably about the target rather than the data itself. Those studies classify disease cross-sectionally, comparing CN against AD; we aim at future conversion, and on top of that they run feature selection without the leakage controls we keep. Signal that holds for cross-sectional classification does not obligingly carry over to predicting future conversion once leakage is controlled, and that is the side of the coin our results speak to.

### 4.4 Methodological contribution

The methodological point of this work is the effect of data leakage, and it is easy to underestimate how large that effect can be. In our own analyses a SOMAscan result moved from a true 0.737 to 0.773 purely because features were selected on the full dataset before cross-validation. That 0.036 is small in absolute terms but misleading in direction, exactly the kind of inflation that erodes trust in biomarker benchmarks. Nested, in-fold feature selection and bootstrap intervals are the cure we report, and the framework is simple enough for other groups to adopt.

### 4.5 Limitations

- Single-center data (ADNI), no external replication cohort; this limits generalizability and constrains the target to a mid-tier journal.
- Sample sizes are modest (*n* = 159 head-to-head; *n* = 183 plasma), and confidence intervals are wide; the marginal CSF gain (+0.03) is not statistically distinguishable from no gain.
- The head-to-head analysis (Table 3) is the only strictly fair comparison; Table 2 compares compartments in different subcohorts and should not be read as head-to-head.
- Inflammation (Olink) showed preliminary signal but with only *n* = 59; it is underpowered and reported as future work, not a result.
- The APOE4 adjustment comparison was underpowered (*n* = 37–39); we therefore do not claim that APOE4 has no interaction with biomarkers.

## 5 Conclusion

A baseline plasma panel supplies most of the predictive information for MCI to AD conversion. That result sits comfortably with the established equivalence of plasma and CSF p-tau217 for AD diagnosis [1], which we carry here into the longitudinal conversion setting and into a head-to-head context that includes multiple omics compartments. The value of this work is chiefly methodological: a single-cohort, leakage-controlled, multi-compartment benchmark that puts a number on the marginal predictive value of plasma, CSF, proteomics, metabolomics, and lipidomics for conversion. Nothing about the framework is specific to AD. The same template applies whenever the question is how much each accessible biological compartment is worth relative to its cost, and that is the trade-off the field keeps needing to quantify.

## Figures and Tables

**Table 1:** Baseline characteristics of the MCI cohort by conversion status.

Characteristic	Converters (n=416)	Non-converters (n=1127)	*p*
Age (years)	74.0 ± 7.1 (n=416)	71.9 ± 7.9 (n=1057)	< 0.001
Education (years)	16.0 ± 2.7 (n=414)	16.0 ± 2.7 (n=1072)	0.892
Plasma p-tau217 (pg/mL)	4.1 ± 3.3 (n=60)	1.6 ± 1.5 (n=123)	< 0.001
Plasma NfL (pg/mL)	5.1 ± 3.2 (n=62)	4.4 ± 4.5 (n=130)	0.240
Plasma GFAP (pg/mL)	3.7 ± 19.9 (n=62)	8.1 ± 40.3 (n=130)	0.311
Plasma A*β*42/40 ratio (Simoa)	0.1 ± 0.0 (n=60)	0.1 ± 0.0 (n=123)	< 0.001
Plasma A*β*42 (MS, pg/mL)	862.8 ± 385.2 (n=158)	1305.4 ± 628.2 (n=297)	< 0.001
Plasma A*β*40 (MS, pg/mL)	8207.1 ± 2138.7 (n=158)	8418.3 ± 2507.9 (n=297)	0.346
CSF A*β*42 (pg/mL)	146.6 ± 41.3 (n=231)	187.4 ± 52.7 (n=385)	< 0.001
CSF t-tau (pg/mL)	112.5 ± 57.3 (n=240)	76.3 ± 47.9 (n=379)	< 0.001
CSF p-tau181 (pg/mL)	47.4 ± 25.0 (n=231)	34.0 ± 19.4 (n=385)	< 0.001
Sex — Female	166 (39. 9%)	489 (43.5%)	0.003
APOE4 — 0 *ϵ*4 alleles	20 (45.5%)	143 (57.0%)	—
APOE4 — 1 *ϵ*4 allele	19 (43.2%)	86 (34.3%)	—
APOE4 — 2 *ϵ*4 alleles	5 (11.4%)	22 (8.8%)	—

**Table 2:** Compartment Benchmark: MCI to AD conversion.

Compartment	Type	n	Events	AUC (95% CI)
C2 Plasma classic	clinical	183	60	0.785 (0.71–0.85)
C8 CSF proteomics (SOMAscan)	omics	417	182	0.758 (0.71–0.80)
C3 Plasma MS A*β*	clinical	455	158	0.748 (0.70–0.79)
C4 CSF classic	clinical	627	248	0.747 (0.71–0.79)
C7 CSF metabolomics	omics	388	165	0.629 (0.58–0.68)
C6 Plasma lipidomics (Duke)	omics	782	350	0.603 (0.56–0.65)
C1 Demographics	clinical	1469	414	0.575 (0.54–0.61)
C5 Plasma metabolomics (Nightingale)	omics	823	341	0.512 (0.47–0.56)

**Table 3:** Marginal-gain analysis of nested models.

Model	AUC (95% CI)	ΔAUC vs previous	NRI
M1 Demographics	0.578 (0.47–0.68)	—	—
M2 + Plasma classic	0.769 (0.68–0.85)	+0.19	+0.79
M3 + CSF classic	0.802 (0.72–0.87)	+0.03	+0.42

## Data Availability

All data used in this study were obtained from the Alzheimer’s Disease Neuroimaging Initiative (ADNI) database (adni.loni.usc.edu) and are available to qualified researchers upon registration and application approval.
